# A Rare Case of Complete Perforation of Endometrial Tissue Through the Mucosa of the Sigmoid Colon

**DOI:** 10.7759/cureus.39038

**Published:** 2023-05-15

**Authors:** Kenny H Do, Jenifer T Do, Ren Y Zhang, M.D.

**Affiliations:** 1 Surgery, Kirk Kerkorian School of Medicine at UNLV (University of Nevada, Las Vegas), Las Vegas, USA; 2 Biology, UNLV (University of Nevada, Las Vegas), Las Vegas, USA; 3 Colorectal Surgery, Nevada Surgery and Cancer Care, Las Vegas, USA

**Keywords:** genetic screening, deep infiltrating endometriosis (die), rectosigmoid colon, colorectal cancer, intestinal endometriosis

## Abstract

Endometriosis is a disease that causes endometrial tissues to proliferate outside of the uterus. The condition is often attributed to estrogen imbalance and can lead to severe inflammation and bleeding, where it is believed that 10% of female patients experience this illness. Endometrial growth can occur in the ovaries, fallopian tubes, stomach, and gastrointestinal tract. Twelve percent of endometriosis cases can be seen in the intestines, with the rectosigmoid colon accounting for 72% of these cases. Patients with intestinal endometriosis may present with moderate symptoms, such as constipation, but they may experience more serious complications as well such as intestinal bleeding. Although the presence of endometrial tissue in the colon is already a rare phenomenon, it is even rarer for endometrial growth to perforate the entire mucosa of the sigmoid colon. A study in 2010 reported that only 21 of such cases have occurred since 1931. The patient in this case report had a gene (MUTYH) mutation that put her at risk for colorectal cancer, and she was ultimately treated with segmental resection of the sigmoid colon. The final pathology of the specimen revealed that the patient’s lesion was endometrial growth. In this case report, we present a rare finding of endometrial tissue perforating through a patient’s intestinal lumen, which was successfully treated with surgical intervention.

## Introduction

Endometriosis is a disease that affects 10% of female patients, where women who have this ailment experience endometrial cellular growth outside of their uterus [1,2]. This physiological process is often caused by abnormal estrogen regulation and can frequently spread to other parts of the peritoneal cavity [1,2]. Besides the uterus, endometrial cells can proliferate in the ovaries, fallopian tubes, bladder, stomach, and intestines. Deep infiltrating endometriosis can also occur when endometrial cells penetrate deeper layers of the targeted organs [2].

Past studies have suggested that 12% of endometriosis cases can be found in the intestines, where the rectosigmoid region accounts for 72% of these intestinal endometrial cases [3]. Despite the common presence of endometrial growth in the intestines, it is very uncommon for the disease to penetrate through the mucosa of the intestinal wall. Although the exact prevalence of full endometrial penetration of the intestines is unclear, one study found that 4.8% of patients in their sample size experienced this kind of deep infiltrating endometriosis in the colon [3]. We believe that the actual prevalence and incidence of endometriosis through the intestinal wall is lower in the general population. Because of the unusual nature of this case, we present a patient who had a resection of her rectosigmoid colon after endometrial growth was seen in the intestinal lumen.

## Case presentation

A 58-year-old female visited a colorectal surgeon after completing an over-the-counter genetic testing assay, called MyRisk Hereditary Cancer Test (Myriad Genetics, Salt Lake City, Utah), which suggested she had a higher risk for a genetic mutation associated with colorectal cancer. The test identified a MUTYH mutation in the patient’s genetic results, a gene that is often associated with colorectal cancer due to its role in base excision repair processes [4].

The patient did not have a family history of colorectal cancer. However, she did have a history of breast cancer. At baseline, the patient did not experience any symptoms such as abdominal pain, bleeding, and changes in bowel habits. She was postmenopausal as well. Her last colonoscopy was performed in 2018.

**Figure 1:**
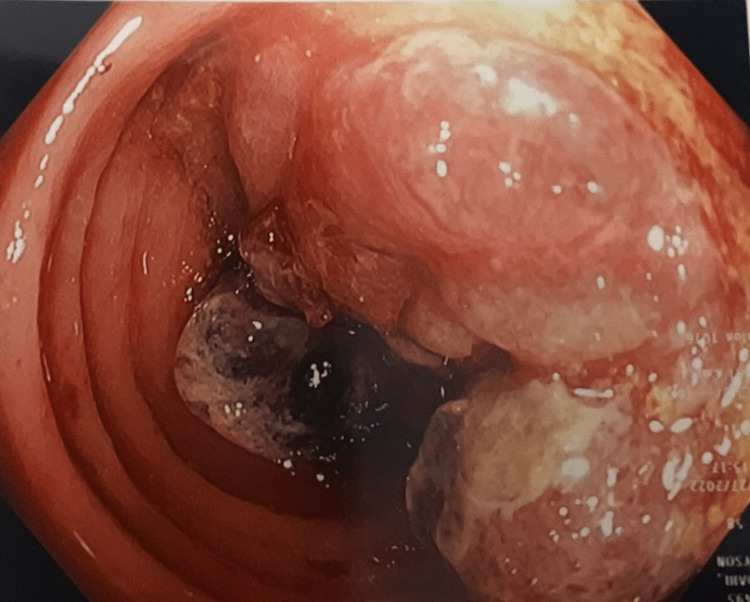
Colonoscopy revealed the presence of a mass in the patient's sigmoid colon

During her consult, the patient reported that she wanted to reduce as much cancer risk as possible. The doctor and the patient agreed to perform a colonoscopy to screen her for any abnormal masses in her colon. She was placed under anesthesia and an endoscopy examination was done on her left lateral decubitus position. No anal canal aberrations were found during the digital rectal examination. During the examination of her colon mucosa, a substantial appendage was detected in the sigmoid colon that was suspicious for malignancy (Figure 1). A tattoo injection was applied to the growth and the mass was taken for biopsy. No abnormal masses were observed in other parts of the patient’s colon and rectum. The adnexa was clear of any lesion bilaterally as well.

The colonoscopic biopsy indicated the presence of tubular adenomas. Based on this finding, a long discussion was done between the surgeon and the patient. They decided on segmental resection of the sigmoid lesion.

**Figure 2:**
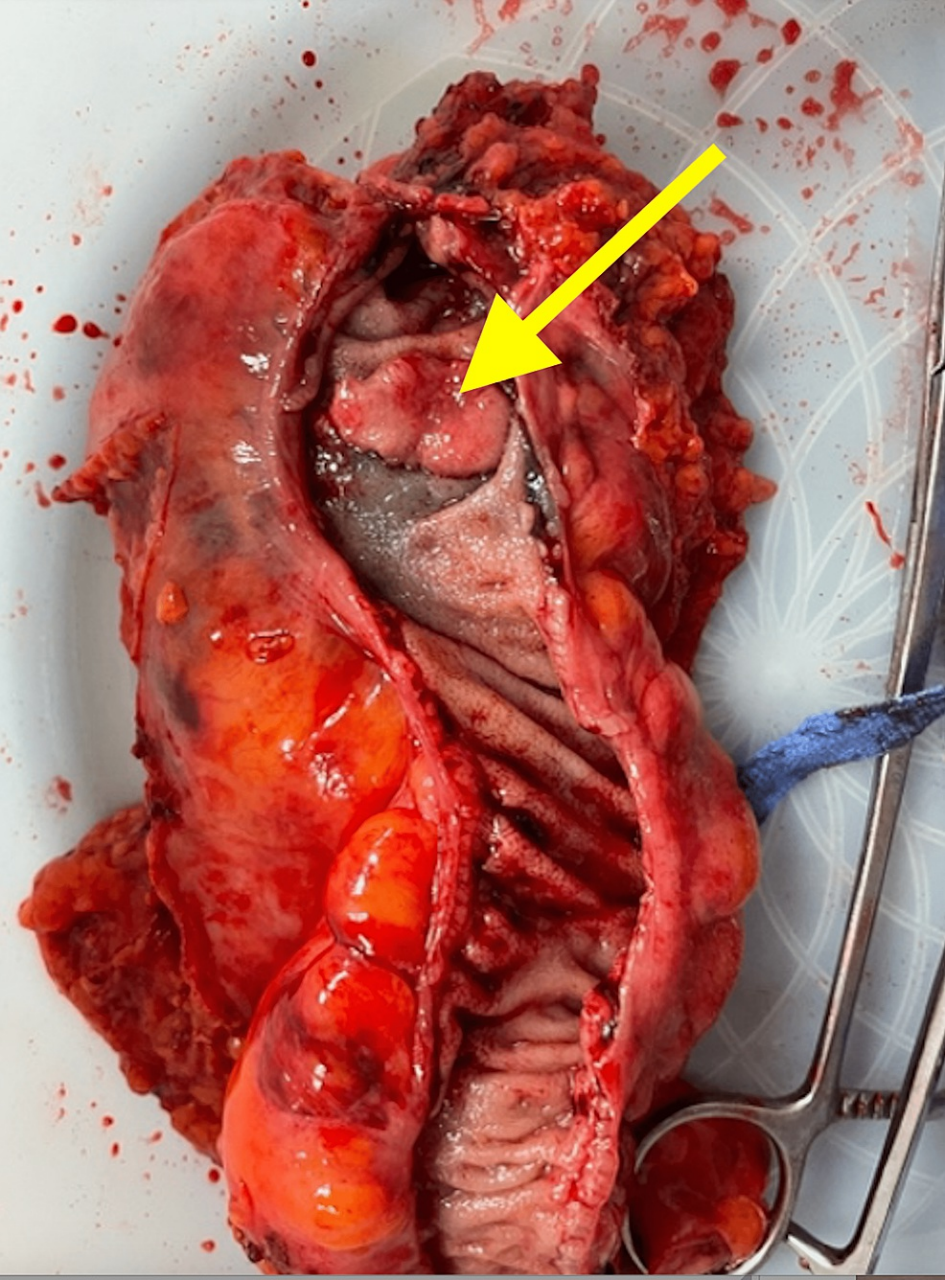
Gross surgical pathology revealed an intestinal endometrial mass of dimensions 2.2 cm X 1.8 cm X 0.4 cm

After discussion and consent, the patient was taken to the operating room for laparoscopic sigmoid resection with end-to-end anastomosis. The postoperative recovery was unremarkable. The gross pathology showed a mass with dimensions of 2.2 cm X 1.8 cm X 0.4 cm (Figure 2). The final pathology report revealed endometriosis penetrating full thickness into the colon mucosa. The specimen did not demonstrate signs of high-grade dysplasia or invasive carcinoma.

## Discussion

The presence of endometriosis in the gastrointestinal tract is not a common condition. When there is endometrial growth in the colon, it usually involves itself within the intramural or serosa layers of the intestines [5]. However, when this rare phenomenon does occur, the endometriosis usually does not penetrate through the entire intestinal wall [3]. As a result, full penetration of the intestinal mucosa is an even rarer condition. This condition of full endometrial penetration of the intestinal mucosa was first published in 1931, where a study from 2010 later reported that only 21 of such cases have been reported in the literature, with 12 of those cases involving the sigmoid colon [6].

Patients who have intestinal endometriosis may experience nonexistent or mild symptoms like the patient in our case report [7]. Patients may also experience bloating, constipation, and uncomfortable bowel movement [7]. However, some of these patients may encounter more serious symptoms such as blockage of the intestines and bleeding in the gastrointestinal tract [6].

The diagnosis of intestinal endometrium first begins with a physical examination, where a colonoscopy, ultrasound, MRI, and other forms of imaging may be incorporated into the diagnosis as well [3]. It is often difficult to diagnose endometrial growth on the bowels because the pathology often appears similar to colon cancer, colon adenoma, colitis, inflammatory bowel disease, and other gastrointestinal diseases [8]. According to the literature, resection of the intestinal segments is usually performed to remove the endometrial perforation of the bowels if cancer is suspected. This method is the most definitive way to rule out other differentials and to identify that the mass is endometrial growth, as endometriosis on the colon often involves the hemosiderin, stroma, and glands of endometrial tissue [8]. Hormone supplements may be given alongside intestinal resection for patients who are interested in having children [5]. Other options may include an oophorectomy or a hysterectomy if the patients are not interested in becoming pregnant [5]. However, for less severe endometriosis that does not perforate the intestines, segmental resection of the bowel may not be necessary. Instead, disc resection and rectal shaving may be sufficient alternatives [7].

## Conclusions

Full penetration of endometrial tissue into the mucosa of the sigmoid colon has a very low prevalence rate. As a result, it is essential that physicians recognize the pathology and symptoms of the disease although it can be hard to differentiate it from other common gastroenterology conditions. To identify and treat deep infiltrating endometriosis, it is important that a full history is taken from the patient. Because our patient had a genetic mutation associated with colon cancer, we decided that it was best to resect the lesion from her sigmoid colon.
